# Online education at the medical School of Tongji University during the COVID-19 pandemic: a cross-sectional study

**DOI:** 10.1186/s12909-021-02951-x

**Published:** 2021-09-28

**Authors:** Yaxiang Song, Shu Wang, Yixian Liu, Xinying Liu, Ai Peng

**Affiliations:** Center for Nephrology and Clinical Metabolomics and Division of Nephrology and Rheumatology, Shanghai Tenth People’s Hospital, Tongji University School of Medicine, Shanghai, 200072 People’s Republic of China

**Keywords:** Medical students and teachers, COVID-19, Online teaching, Questionnaire, Prospective study

## Abstract

**Background:**

The global reputation of coronavirus disease (COVID-19) has led universities in China to conduct online teaching. However, the actual feedback from medical teachers and students regarding online education remains unclear.

**Methods:**

A prospective questionnaire survey examined the current opinions of online education from teachers and students at the Medical School of Tongji University.

**Results:**

A total of 488 valid questionnaires were collected (223 males, 45.7%; 265 females, 54.3%), including 394 students (80.7%) and 94 teachers (19.3%). Most teachers and students were “in favor of online teaching,” had “positive views for online education,” were “satisfied with online teaching,” and “expected for regular online education,” although students thought that “too much learning tasks had been assigned” (90.4% teachers vs. 43.1% students, *P* < 0.001) and “less teaching effect than in offline classes” (68.1% teachers vs. 43.4% students). Compared to female counterpart, male students had higher “learning interest” (27.6% vs. 14.9%), “learning attention” (29.2% vs. 14.4%), “learning efficiency” (30.2% vs. 16.7%), and “better learning effect” (27.6% vs. 15.3%). Furthermore, male students had a significantly rise in attendance rate. Compared with male teachers, female teachers had less “experience in online educational course recording” (25.9% vs. 50%) and “past training for online teaching” (53.7% vs. 77.5%). Furthermore, they tended to be more “resistant to online teaching” (44.4% vs. 22.5%) and less “ready for online teaching” (70.4% vs. 87.5%). There was no significant difference in the acceptance of online teaching among teachers in different age groups.

**Conclusions:**

Most teachers and students supported and were satisfied with the implementation of online education during the pandemic. Although teachers were less adaptable to online education, they still had positive opinions. Sex influenced the acceptance of online teaching. Male teachers and students showed better adaptability than their female counterparts. Although online teaching has advantages, it still cannot completely replace traditional offline teaching. As online education is a trend for future learning, universities should make more efforts to improve it, especially to provide more attention to female teachers and students.

## Background

The coronavirus disease of 2019 (COVID-19), transmitted mainly through the respiratory tract, has spread globally, and it has high morbidity and mortality [1]. To control the pandemic, the Chinese government has implemented strict prevention measures, such as prohibiting gatherings, closing schools, isolating people at home, and maintaining social distance [2]. Unlike face-to-face teaching, online classes are not restricted by geographic regions, so teachers and students can complete teaching tasks at home. The online teaching mode is thought to be an effective way in reducing the spread of infectious diseases in colleges by breaking chains of transmission [3, 4]. Hence, Chinese colleges and universities have adopted online teaching practices [5].

Diversified online education can enrich learning and teaching methods [6, 7]. Teaching management can be completed through network interactions, which are efficient and convenient [8, 9]. Moreover, students could watch recordings of classes repeatedly offline. However, online teaching might weaken the supervision of students [10], and it is more common for students to skip classes and follow the lessons less carefully [10, 11]. Besides, Mental health is another major problem [12]. Home segregation will cause anxiety and irritability due to limitation of activity space, lack of outside entertainment, worrying about health, and uncertainty about the future [13, 14]. Therefore, as a new mode of teaching, online education still requires more research.

Online education in colleges and universities has been compulsory during the COVID-19 pandemic. However, the actual feelings and opinions of teachers and students are still unclear. Therefore, it is difficult for university administrators to take effective measures to improve it and guarantee the teaching effect. Thus, we investigated the status of online teaching in the medical school of Tongji University during the pandemic via an online questionnaire survey.

## Methods

An online survey was conducted from March 24, 2020, to April 10, 2020. Self-designed questionnaires were distributed to teachers and students at the Medical School of Tongji University and were to be completed anonymously. All participants were aged > 18 years. The teachers and students received the questionnaire via their social software accounts and were asked to complete it in 2 weeks, with repeated reminders during the valid period.

The questionnaire contained a total of 43 questions. Data were collected on age, sex, psychological state, opinions of online teaching, learning and teaching effects, and suggestions for online teaching in the future.

Questionnaire data with too short a response time (< 2 min) were excluded, as these questionnaires were thought completed without thinking and the data were unreliable. The same person could answer the questionnaire only once. All the data were imported into SPSS 24.0, and analyzed using the chi-squared test.

## Results

### General information

A total of 490 subjects responded, which included 488 valid questionnaires with a response rate of 100% and an effective rate of 99.6%. There were 223 males (45.7%) and 265 females (54.3%), including 394 students (80.7%) and 94 teachers (19.3%). Among all the teachers, 39 (41.5%) were aged < 40 years. There was no statistical difference in the age ratio between male and female teachers, or in the number of students between sexes.

### Comparison of teachers’ and students’ opinions on online education

There were no statistical differences between teachers and students in such aspects, including “in favor of online teaching,” “positive views for online education,” “satisfaction with online teaching,” “expectation for regular online education,” and “in favor of resource sharing among colleges.” However, there were statistically significant differences in responses to questions such as “good communication in online class” (47.9% teachers vs. 29.7% students, *P* < 0.05), “good adaptation to online teaching” (81.1% teachers vs. 89.8% students, *P* < 0.05), “too much learning tasks assigned than in offline teaching” (90.4% teachers vs. 43.1% students, *P* < 0.001) and “ideal teaching effect of online education” (68.1% teachers vs. 43.4% students, *P* < 0.05) (Table 1).

**Table 1:** Comparison of opinions on online teaching between teachers and students

Question	Identity		Total (%) *N* = 488	*P-*value
	Student (%) *N* = 394	Teacher (%) *N* = 94		
In favor of online teaching	353 (89.6)	82 (87.2)	435 (89.1)	0.509
The views for online education				
Only an emergency measure	155 (39.3)	37 (39.4)	192 (39.3)	0.999
Supplement to offline teaching	189 (48.0)	50 (53.2)	239 (49.0)	0.363
Replacement of offline teaching	27 (6.9)	5 (5.3)	32 (6.6)	0.59
Good adaptation to online teaching	354 (89.8)	77 (81.1)	431 (86.5)	0.017
Good communication in online class	117 (29.7)	45 (47.9)	162 (33.2)	0.001
Too much learning tasks assigned than in offline teaching	173 (43.1)	85 (90.4)	258 (52.8)	< 0.001
Ideal teaching effect of online education	171 (43.4)	64 (68.1)	235 (48.2)	< 0.001
Satisfaction with online teaching	243 (61.7)	53 (60.7)	209 (61.5)	0.625
Expectation for regular online education	114 (28.9)	25 (26.6)	139 (28.5)	0.652
In favor of resource sharing among colleges	246 (62.4)	66 (70.2)	312 (63.9)	0.158

### Comparison of opinions on online learning between male and female students

Regarding online education, male students had a much greater increase in attendance rate (20% vs. 10.1%), learning interest (27.6% vs. 14.9%), and learning attention (29.2% vs. 14.4%). In addition, male students had higher learning efficiency (30.2% vs. 16.7%), better learning effect (27.6% vs. 15.3%), more worries about study delay (82.5% vs. 73.5%), and a greater sense of identity for too many learning tasks (54.6% vs. 34.6%). There was no significant difference in the following aspects: “in favor of online teaching,” “good communication in online learning,” “satisfaction with teaching quality,” “less exercise during online teaching,” “irregular daily schedule,” “anxiety moods,” “spending extra time on electronic devices,” and “slack in online class” (*P* > 0.05) (Table 2).

**Table 2:** Learning effect of students regarding online learning (male vs. female)

Item	Sex		Total (%) *N* = 394	*P*-value
	Male (%) *N* = 183	Female (%) *N* = 211		
The most concerned questions				
Delay in study	151 (82.5)	155 (73.5)	306 (77.7)	0.031
Anxiety moods	132 (72.1)	144 (68.3)	276 (70.1)	0.401
Irregular daily schedule	83 (45.4)	97 (46.0)	180 (45.7)	0.903
Less exercise	74 (47.0)	89 (43.6)	163 (41.4)	0.726
In favor of online teaching	158 (86.3)	195 (92.4)	353 (89.6)	0.791
Spending extra time on electronic devices				
1–3 h	46 (24.9)	46 (22.0)	92 (23.4)	0.504
3–5 h	77 (41.6)	77 (36.9)	154 (39.1)	0.332
5–7 h	39 (21.1)	56 (26.8)	95 (24.1)	0.186
7–9 h	11 (6.0)	20 (9.6)	31 (7.9)	0.182
More time	12 (6.5)	10 (4.8)	22 (5.6)	0.463
Attendance rate				
Reduced	24 (13.0)	11 (5.2)	35 (8.9)	0.007
Unchanged	122 (67.0)	179 (84.7)	301 (76.4)	< 0.001
Rose	37 (20.0)	21 (10.1)	58 (14.7)	0.005
Slack in online class	63 (34.1)	67 (32.1)	130 (32.8)	0.674
Good communication in online class	59 (23.3)	58 (27.5)	117 (29.7)	0.303
Better learning effect than offline education	51 (27.6)	32 (15.3)	83 (21.1)	0.003
Learning interest in online class				
Rose	51 (27.6)	31 (14.9)	82 (20.8)	0.002
Unchanged	90 (48.7)	124 (59.3)	214 (54.3)	0.034
Decreased	44 (23.7)	54 (25.8)	98 (24.9)	0.638
Learning attention in online class				
Rose	54 (29.2)	30 (14.4)	84 (21.3)	< 0.001
Unchanged	61 (33.0)	97 (46.4)	158 (40.1)	0.007
Decreased	70 (37.9)	82 (39.2)	152 (38.6)	0.776
Better learning efficiency in class	56 (30.2)	35 (16.7)	91 (23.1)	0.001
Too much learning tasks	100 (54.6)	73 (34.6)	173 (43.1)	< 0.001
Satisfaction with teaching quality	125 (68.3)	118 (55.9)	243 (91.7)	0.051

### Views of teachers across different sex and ages

Female teachers had a lower proportion of votes regarding the following aspects: “resistance to online teaching” (44.4% vs. 22.5%), “ready for online teaching” (70.4% vs. 87.5%), “past record experience” (25.9% vs. 50%), “past training for online teaching” (53.7% vs. 77.5%), and “sparing more time on preparing online class” (59.3% vs. 92.5%) (*P* < 0.05). There was no significant difference in each question between teachers older or younger than 40 years old (*P* > 0.05) (Table 3).

**Table 3:** Online teaching status of teachers

Item	Age Group		*P*-Value	Sex		*P*-Value
	< 40 (%) *N* = 39	≧40 (%) *N* = 55		Male (%) *N* = 40	Female (%) *N* = 54	
Resistance to online teaching	8 (20.5)	5 (9.1)	0.106	9 (22.5)	24 (44.4)	0.031
Past record experience	14 (35.9)	20 (36.4)	0.963	20 (50.0)	14 (25.9)	0.016
Awareness of the meaning of online teaching	23 (59.0)	37 (67.3)	0.409	30 (75.0)	30 (55.6)	0.052
Ready for online classes	30 (76.9)	43 (78.2)	0.885	35 (87.5)	38 (70.4)	0.049
Past training for online teaching	26 (66.7)	34 (61.8)	0.63	31 (77.5)	29 (53.7)	0.018
Adaptation to the online teaching	30 (76.9)	47 (85.5)	0.29	36 (90.0)	41 (75.9)	0.08
Sparing more time in preparing online class	29 (74.4)	40 (72.7)	0.86	37 (92.5)	32 (59.3)	< 0.001
No influence on teaching enthusiasm	26 (66.7)	40 (72.7)	0.527	29 (72.5)	37 (68.5)	0.585
Achievement of ideal goals	27 (69.2)	36 (83.6)	0.701	30 (75)	33 (61.1)	0.127

### Suggestions for future online teaching

Teachers hoped to improve the function of live broadcast software (70.2%), strengthen the management of online learning (69.2%), make full use of current online resources (55.3%), and upload supporting educational materials in time for the reference (34.0%). Meanwhile, students hoped to optimize learning software (75.1%), enhance online classroom experience (61.9%), and raise learning consciousness (52.8%). Both teachers (67.0%) and students (59.1%) anticipated constructing a resource-sharing system of online teaching among different schools.

## Discussion

Online teaching was one of the Chinese government’s initiatives to prevent and control the spread of COVID-19. Our research showed that the majority of teachers and students in medical schools supported online teaching during the pandemic. This may be explained by the following findings: (1) online teaching was able to effectively prevent the infection of infectious diseases [3, 15]; (2) all the students and teachers were satisfied with and in favor of the government’s anti-epidemic work [16, 17]; (3) online teaching could make the university teaching work advance as scheduled [18]; and (4) online teaching could relieve the anxiety of medical students about quarantine and exert a positive effect on their mental health [19, 20].

In the process of online teaching, most teachers and students could adapt to changes in teaching forms. Although teachers were relatively less capable of acclimatizing themselves to online teaching, they still believed online mode had an ideal teaching effect, which was higher than that of the student group. Because of intellectual deterioration and cognitive decline due to aging, elderly people may be slower to accept new things [21–23]. However, teachers are more experienced and could be aware of the online teaching shortcomings and may accurately estimate its effect.

Interestingly, there were significant differences in the adaptability and learning status of online teaching between males and females in both teachers and students (*P* < 0.05). Thus, male teachers tended to be more adaptable to online teaching. Compared to female students, male students had a higher increase in learning interest, learning attention, attendance rate, and learning satisfaction. Males are generally more familiar with computers and are more easily adaptable to online teaching [24]. Females usually have more delicate emotions and place higher demands on themselves, which makes them more prone to stress [25] and feeling worried [26]. These personalities and characteristics pertaining to sex may partly explain these differences.

Interestingly, in the present study, we found that many more teachers thought online education had made them assign too many learning tasks. The reasons may be described as follows: (1) poor online communication between teachers and students [27]; (2) in teachers’ opinion, students might have relatively low learning efficiency during home isolation [28, 29]; and (3) lack of initiative of students on learning due to learning more independently than offline education [30]. Therefore, during the epidemic period, while improving students’ learning autonomy, teachers should try to communicate more with students and coordinate the amount of learning tasks.

Our study showed that the advantages of online education for medical students included the following: (1) reducing the risk of COVID-19 infection caused by population flow, while making teaching tasks be carried out during quarantine; (2) through course playback and courseware sharing, students can flexibly arrange their study time. However, the disadvantages of online teaching included the following: (1) objective long distance limited the interaction between teachers and students, influenced teachers’ personal style and students’ attention in class, and made the students’ learning more difficult to manage; and (2) long-term use of electronic products could damage eyesight and health. Hence, online teaching cannot completely replace traditional teaching as a temporary alternative. These findings were similar to those in online teaching studies conducted in other majors [31], which indicates that the combination of online and offline teaching may become a new normal for medical education in the future.

Due to the short preparation time and heavy teaching tasks, there were many problems in the implementation of online teaching during the epidemic. The following solutions might be helpful: (1) making full use of the uploaded online courses to avoid wasting teaching resources by duplication of recording; (2) optimizing and integrating learning software to improve online learning experience; (3) strengthening the interaction between teachers and students in online courses; and (4) giving more attention to females, such as providing more training opportunities for online teaching to female teachers, enhancing the interest and enthusiasm of female students in online classes, and so on.

### Limitations

First, all our questionnaire respondents majored in clinical medicine; therefore, the conclusion only represents their subjective feedback on online teaching. Second, our survey relied on respondents’ online self-reporting. Although it is convenient to collect data, the subjective judgment of respondents may lead to recall bias and misclassification. To this end, we guaranteed anonymity and confidentiality to all interviewees to exclude untrue data as much as possible.

## Conclusions

Most teachers and students in medical schools supported and were satisfied with the implementation of online teaching during the pandemic. Although teachers were less adaptable to online teaching than students, they still held positive attitude toward it and believed online teaching was more effective. Sex differences significantly affected the acceptance of online teaching. Although online teaching has unique advantages such as breaking the space limit and making learning time more flexible, its inherent defects of objective distance and less direct communication make it impossible to replace traditional teaching at present. However, online education is still a trend for future learning, and universities should make more effort to improve it.

## Data Availability

All data generated or analyzed during this study are included in this published article.
